# Modelling the potential for soil carbon sequestration using biochar from sugarcane residues in Brazil

**DOI:** 10.1038/s41598-020-76470-y

**Published:** 2020-11-10

**Authors:** David Lefebvre, Adrian Williams, Jeroen Meersmans, Guy J. D. Kirk, Saran Sohi, Pietro Goglio, Pete Smith

**Affiliations:** 1 School of Water, Energy and Environment, Cranfield University, College Road, Bedford, MK43 0AL UK; 2 TERRA Teaching and Research Centre, Gembloux Agro-Bio Tech, University of Liège, 5030 Gembloux, Belgium; 3 UK Biochar Research Centre (UKBRC), School of GeoSciences, University of Edinburgh, Crew Building, Edinburgh, EH9 3FF UK; 4 Wageningen Economic Research, Wageningen University & Research, Leeuwenborch, Hollandsweg 1, 6706KN Wageningen, The Netherlands; 5 Institute of Biological and Environmental Sciences, University of Aberdeen, 23 St Machar Drive, Aberdeen, AB24 3UU UK

**Keywords:** Climate-change mitigation, Computational models, Carbon cycle

## Abstract

Sugarcane (*Saccharum officinarum* L.) cultivation leaves behind around 20 t ha^−1^ of biomass residue after harvest and processing. We investigated the potential for sequestering carbon (C) in soil with these residues by partially converting them into biochar (recalcitrant carbon-rich material). First, we modified the RothC model to allow changes in soil C arising from additions of sugarcane-derived biochar. Second, we evaluated the modified model against published field data, and found satisfactory agreement between observed and predicted soil C accumulation. Third, we used the model to explore the potential for soil C sequestration with sugarcane biochar in São Paulo State, Brazil. The results show a potential increase in soil C stocks by 2.35 ± 0.4 t C ha^−1^ year^−1^ in sugarcane fields across the State at application rates of 4.2 t biochar ha^−1^ year^−1^. Scaling to the total sugarcane area of the State, this would be 50 Mt of CO_2_ equivalent year^−1^, which is 31% of the CO_2_ equivalent emissions attributed to the State in 2016. Future research should (a) further validate the model with field experiments; (b) make a full life cycle assessment of the potential for greenhouse gas mitigation, including additional effects of biochar applications on greenhouse gas balances.

## Introduction

Sugarcane (*Saccharum officinarum* L.) is the world's largest crop by production quantity, with a total of 1.8 billion tonnes of cane produced globally per annum in more than 90 countries^1^. Sugarcane fields were traditionally burned to facilitate manual harvest^2^. However, to avoid air pollution, in many countries the fields are now mostly left unburned and harvested mechanically. This ‘green harvesting’ leaves large quantities of biomass (hereafter referred as ‘trash’) in the field^3^. Although trash provides a mulch that can benefit soil fertility and the growth of subsequent crops, it can also increase the risk of fire, pest proliferation, and reduced soil warming and drying in the spring^2,4^. Currently it is typical for all the trash to be left on the field, although studies into sustainable rates of removal have been made^4,5^. A potential alternative use of the trash is for energy generation, substituting for fossil fuels^6,7^. Another option is to make biochar, which potentially provides greenhouse gas (GHG) removal as well as returning carbon (C) and nutrients to the soil^8,9^. It is also argued that biochar has additional GHG abatement potential through effects on crop production, including reduced requirement for manufactured fertilizer^7^.

Predicting the potential of biochar for these purposes requires allowance for the wide range of biochar types that can be created, and the variable effects of soil conditions on biochar decomposition, and vice versa. The properties of biochar vary according to pyrolysis conditions and other manufacturing parameters as well as the nature of the biomass ‘feedstock’^10,11^. The effects on existing soil organic carbon (SOC) may lead to increased SOC mineralization (‘positive priming’) or decreased mineralization (‘negative priming’)^12,13^. Studies report negative, positive and no priming effect^12–14^, sometimes with a change in the direction of priming over time, typically from increased SOC mineralisation in the first year or so, to decreased mineralization thereafter^12^. In a recent meta-analysis, Wang et al. (2016) found a wide range of priming effects depending on biochar and soil characteristics; but they found consistently large priming effects in low-fertility sandy soils which are typical of many sugarcane areas.

Attempts to model long-term increases in SOC following biochar application have relied on data from short-term studies^15^. Dil and Oelbermann adapted the CENTURY model to evaluate the long-term effect of biochar by representing it as 95% lignin added to the ‘slow C pool’ in CENTURY^16^. However, this pool has a turnover time of 10 to 50 year^17^ which is at least an order of magnitude faster than typical biochar turnover^18^. Archontoulis et al. developed a biochar sub-model for the Agricultural Production System Simulator (APSIM), and its simulations compared favourably with some experimental observations, but it lacked wider calibration and validation^19^. Lychuk et al. developed a sub-model to integrate biochar into the Environmental Policy Integrated Climate (EPIC) model by allowing for the effect of biochar on the initial soil properties represented in the model, but not explicitly for biochar turnover^20^. Mondini et al. modified the RothC SOC model to better describe the decomposition of exogenous organic matter, such as biochar, but without specific modifications for biochar^21^. Overall, none of the existing models of biochar turnover in soils is suitable for our purposes, as these models were not optimized for biochar-amended soils.

In this paper, we develop and test a biochar sub-model for the RothC model with which to assess the potential for excess sugarcane trash and the bagasse residues to produce biochar for in-field soil C sequestration. The sub-model divides the biochar C into fresh plant material, which is fed into RothC, and recalcitrant material, which slowly decomposes to CO_2_; and the combined model predicts long-term changes in SOC as a function of biochar properties and soil, cropping and environmental conditions. We evaluate the model against published data and use it to make predictions for São Paulo State in Brazil using available data on sugarcane production and relevant soil and climate conditions. São Paulo is a suitable case study because Brazil accounts for 40% of global sugarcane production and São Paulo accounts for 55% of the national production^22^ with 96% of its sugarcane fields now mechanically harvested^22,23^. We compare three scenarios in which either 100%, 50% or 25% of the available sugarcane residues are used to produce biochar, with the remaining residues added to the fields as fresh material. Thereby we provide the first assessment of the carbon capture potential of sugarcane biochar at a regional scale, accounting for different climatic and soil characteristics.

## Results and discussion

### Model evaluation

**Figure 1:**
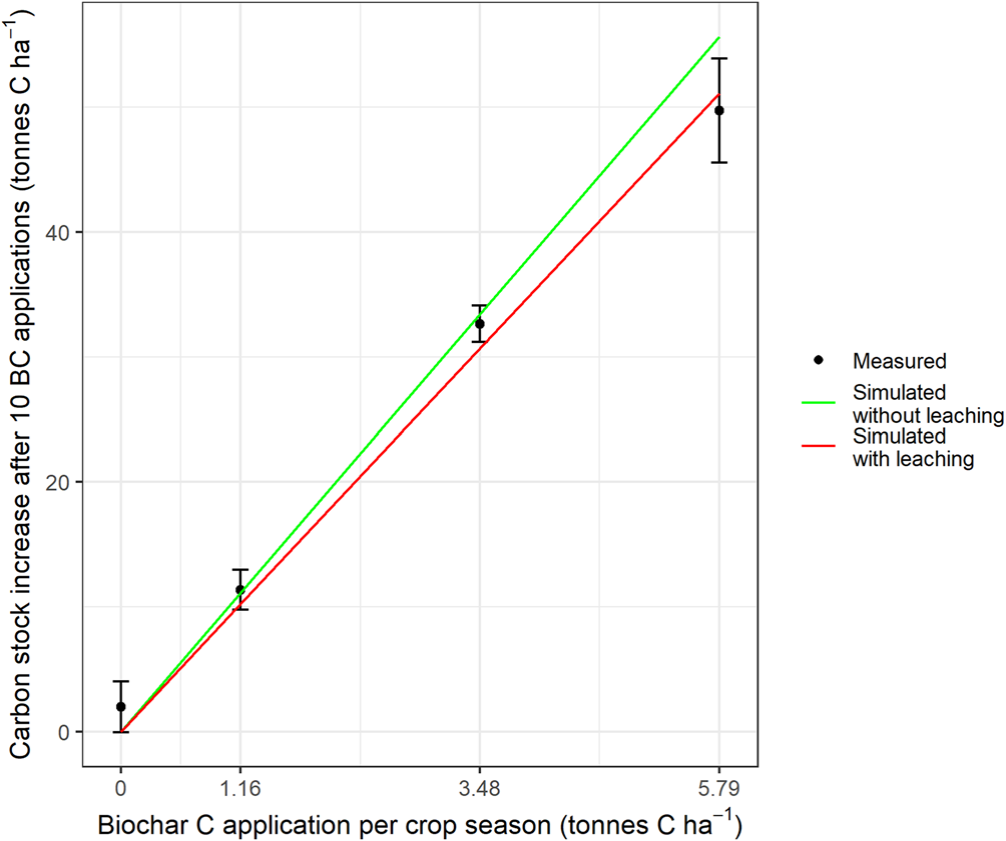
Comparison of the soil C stock increase after 5 years of biochar addition (10 applications of biochar) between measured values (black dots ± SD) and simulated (red and green lines) using the modified RothC model described in this manuscript. The red line accounts for the loss of 5.2% year^−1^ of the biochar via leaching.

Comparison of simulated values against the experimental observations from Liu et al.^24^ is presented in Fig. 1. Accounting for leaching of biochar particles to sub-soil at 5.2% of the biochar per year (“Model evaluation” section in Methods) improved the agreement with the experimental data. Liu et al. reported a loss rate of between 11.8 and 19.1% of the BC-C over the 5 years, estimated by mass balance^24^. Our model reaches a loss of 4% of BC-C after 5 years without leaching and 12% with leaching, with no modelled interaction with application rate.

Additional limited evaluation of the model was possible by comparison with the results of controlled-environment experiments (soil–biochar, plant free, incubation experiments) and the dataset used by Archontoulis et al.^19^ to validate their APSIM biochar sub-model (Supplementary Information). The evaluation results using that prior dataset^19^ (Supplementary Fig. S4) fits with their biochar sub-model validation results. Comparison with incubation experiments showed that our modelled biochar mineralization rate reflects the general trend reported in the literature (Supplementary Fig. S5).

The extent of this evaluation exercise is limited but we consider it the best available at the current time, owing to the general paucity of long-term data. The intensive investigation of biochar is rather recent compared to the heritage of long-established field experiments^25^. The good level of fit provides some confidence in the model predictions.

### Potential C sequestration

**Figure 2:**
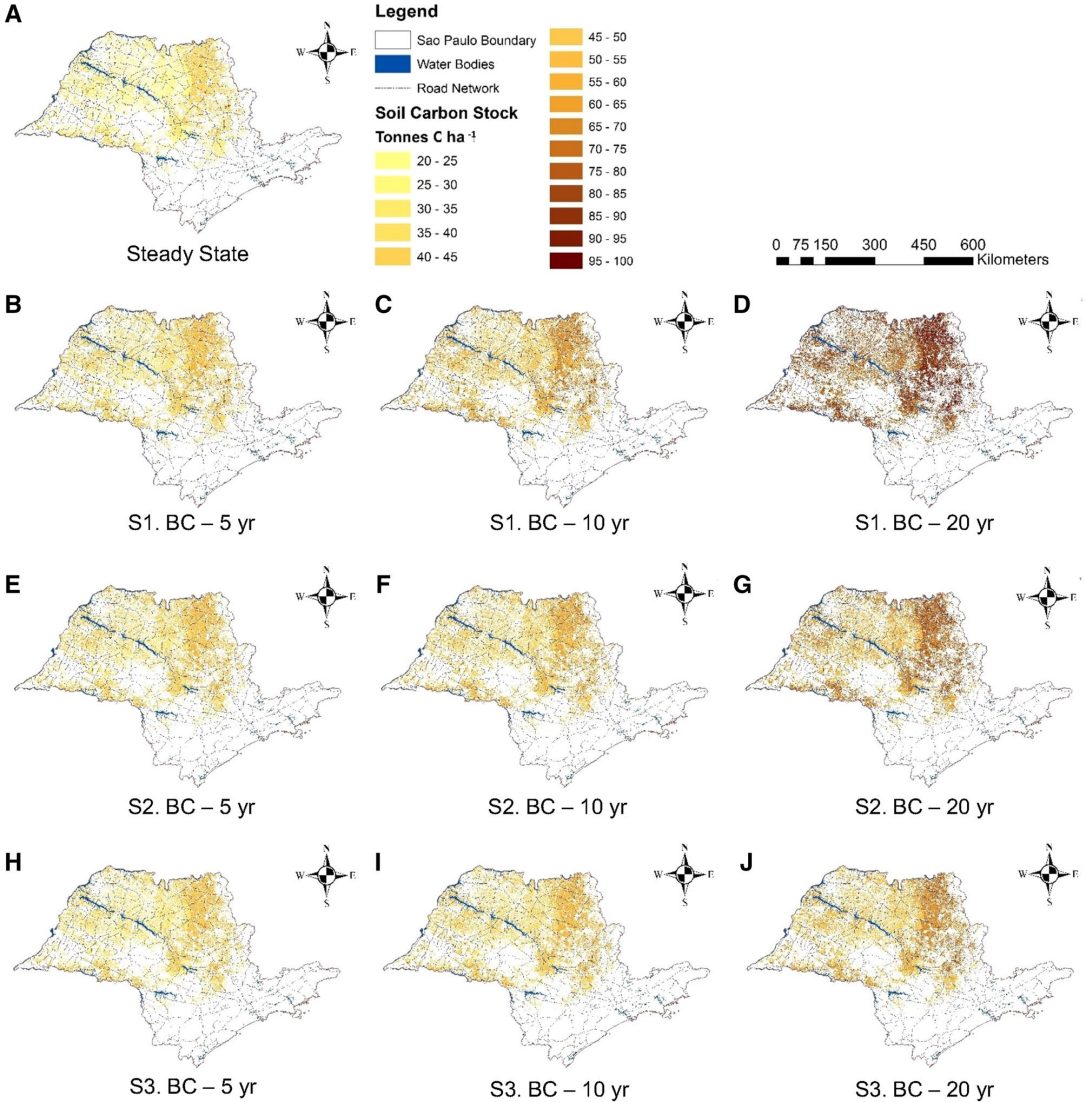
Predicted SOC stock in sugarcane fields across São Paulo. At steady state (**A**) and after 5, 10, and 20 year of biochar addition for Scenarios 1 (**B**,**C**, and **D** respectively), 2 (**E**,**F**,**G**), and 3 (**H**, **I**, **J**). For Scenarios 1, 2, and 3, the biochar additions are 2.46, 1.23, and 0.62 t C ha^−1^ year^−1^ respectively and the fresh C inputs are 6.57, 9.74, and 11.1 t C ha^−1^ year^−1^, respectively as trash, bagasse, roots, root exudates, and applications of vinasse and filter cake.

Figure 2 shows the predicted SOC stocks in sugarcane fields across São Paulo for the three biochar application scenarios (“Modelled Scenarios” section). There is a slightly higher C stock in the north-eastern region of São Paulo (Fig. 2A), consistent with field measurements^26^. This region is characterized by a clayey soil (Rhodic Ferralsols according to the FAO classification^27^ or Ustox according to the USDA classification^28^) and mild climate (Supplementary Table S2). Both characteristics are known to either increase the C storage capacity in soils (i.e. high clay contents)^29^ or decrease the mineralization rate of C in soil (i.e. low temperatures)^30^.

**Figure 3:**
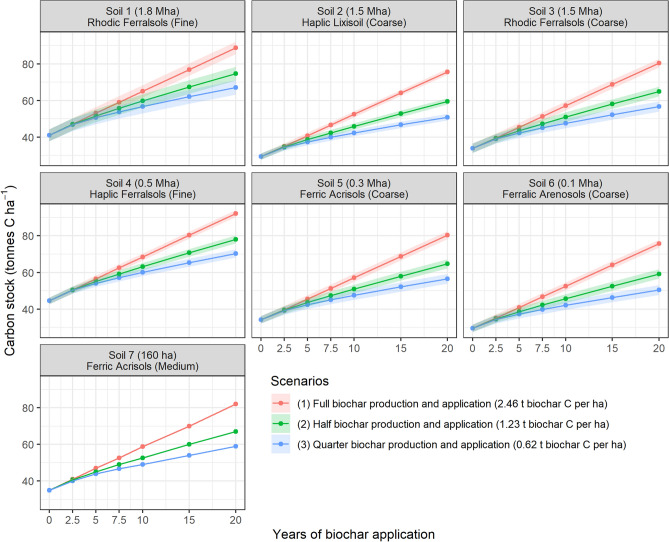
Effect of FAO soil type on predicted SOC stock up to 20 year. Dots are means across sugarcane fields at the indicated times (year 0 is the steady state value); ribbon width is 1 SD due to geographical climate variability; lines indicate general trends and not intermediate points. The SD is small for Soil 7 because they account for only a few sugarcane fields concentrated in one particular region (Supplementary Table S3). The land area associated with each soil type is shown in parenthesis for each plot.

Under Scenario 1 (Fig. 2B–D), there is a steady increase in the SOC stock due to the incremental addition of biochar. Under Scenarios 2 (Fig. 2E–G) and 3 (Fig. 2H–J), there is a similar SOC increase in the first 5 year after biochar application but a lower soil C stock increase after 10 and 20 year as compared to Scenario 1. The reduction at 10 and 20 year indicates that the conventional soil C pools reach new equilibria, while the biochar added in Scenario 1, present in the recalcitrant (RBC) pool, is subject to much slower mineralization and progressively expands.

Figure 3 shows the C stock increase over the three considered periods for each soil type and biochar scenario. The consistently lower C stock at 10 and 20 year for Scenarios 2 and 3 reflects the results shown in Fig. 2. The C accumulation rate decreases over time for both Scenarios 2 and 3 as a new SOC content equilibrium is reached. The increased addition of fresh plant material in Scenario 2 and 3 leads to a sudden increase of decomposable carbon in the soil system, which will eventually supply all carbon pools and affect their transformation rate to reach a new equilibrium. This slow process explains the decreasing rate of soil C stock increase witnessed for Scenario 2 and 3 as, with time, the additional fresh organic matter modelled in these Scenarios will not affect the soil C stock anymore. The overall higher C stocks in Ferralsols is due to their higher clay contents (Supplementary Table S1).

### Priming effects

**Figure 4:**
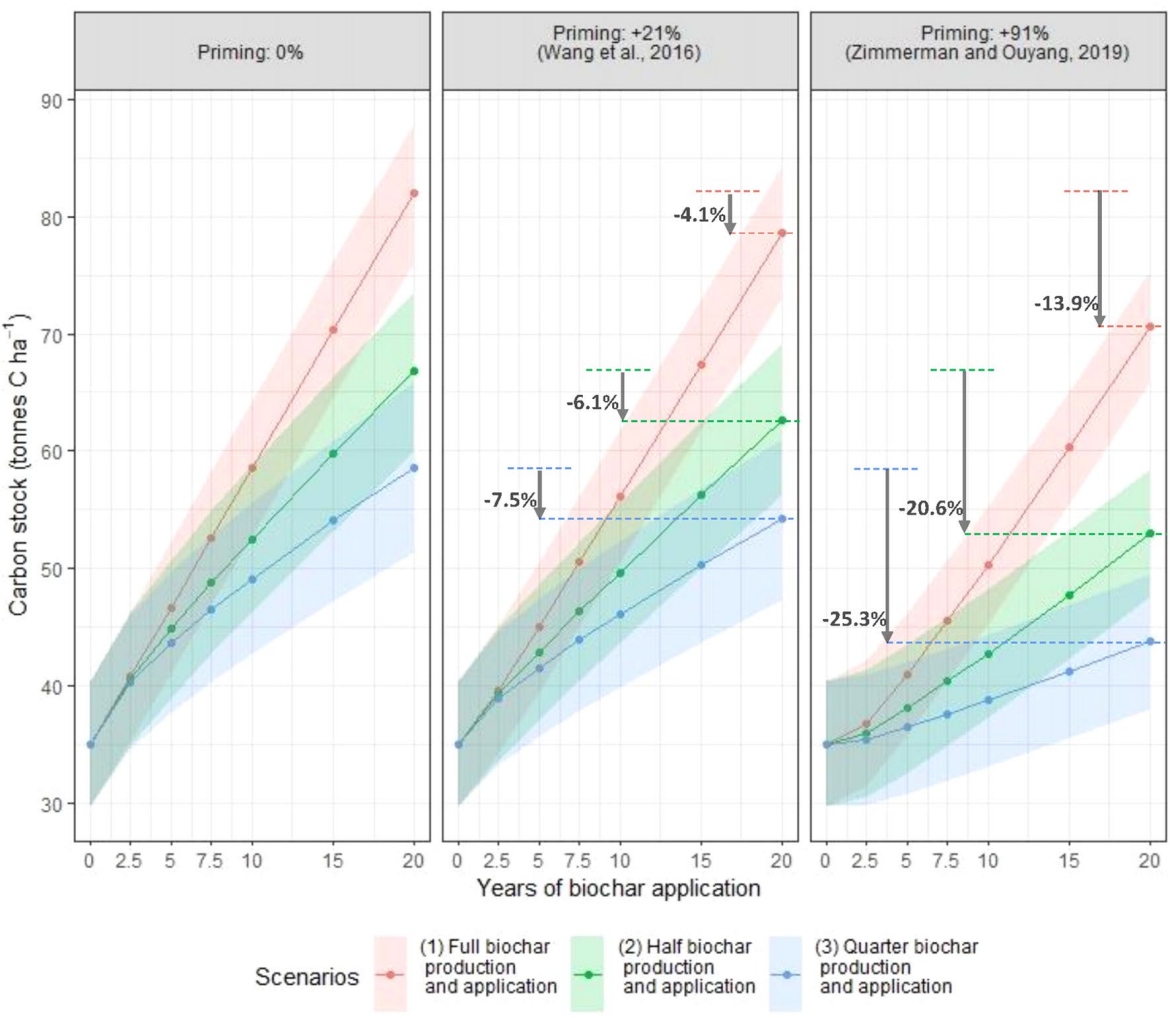
Priming effect of biochar on SOC under the three addition scenarios. Dots are mean values across sugarcane fields at the indicated times (year 0 is the steady state value); ribbon width is due to climate and soil variability (1 SD). The downward arrow indicates the % decrease in SOC stock after 20 year compared to no priming.

Including biochar-induced priming reduces the predicted increases in SOC (Fig. 4). Since priming was modelled independent of dose, the proportional effect on SOC is least when the addition of biochar is highest (Scenario 1). The SOC accumulation relative to baseline was -4.1% using the conservative assumption (SOC decomposition rate increased 21%) and 13.9% for the extreme assumption (SOC decomposition increased 91%). The corresponding effects for Scenario 2 were − 6.1% and − 20.6%. The effects for Scenario 3 were − 7.5% and − 25.3% Scenario 3. Overall, a biochar-induced positive priming effect could impair the C sequestration potential of the practice. However, even under the extreme degrees of priming effect modelled here, the net SOC balance with biochar addition remains positive, resulting in substantial carbon sequestration and CO_2_ removal.

### Sequestration potential of the whole state

**Figure 5:**
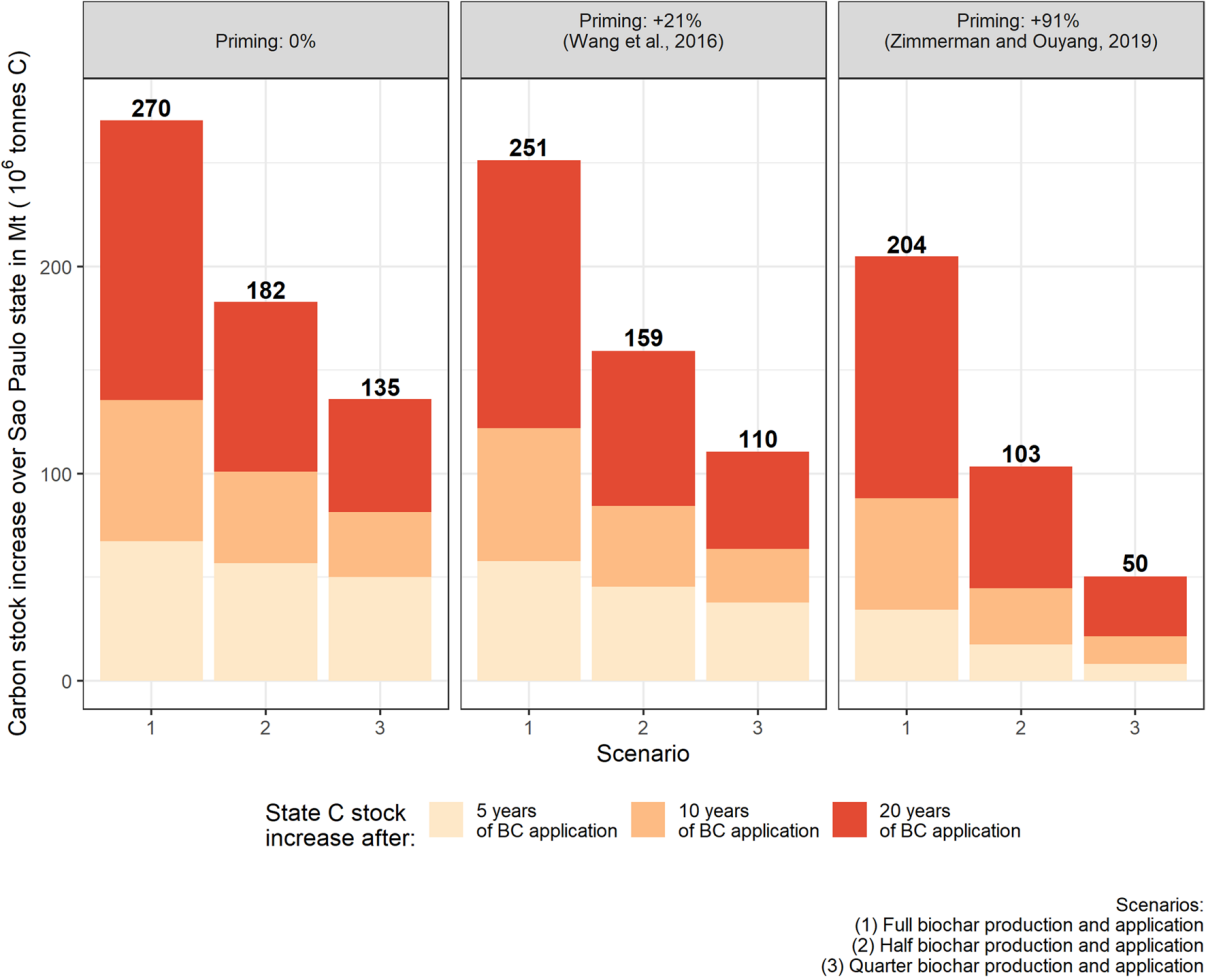
São Paulo State C sequestration potential. The number in bold above each bar represent the State total C sequestered after 20 year of biochar addition in Mt of C (10^6^ t C).

These three scenarios result in a wide range of potential C sequestration over all 5.77 Mha^31^ of sugarcane fields in São Paulo State (Fig. 5). Considering 20 year of biochar application, the most effective scenario in term of C sequestration (Scenario 1, no priming of SOC) could sequester 13.5 Mt of C per year over the State (49.5 Mt of CO_2_e), or 31% of the 159 Mt CO_2_e emissions attributed to the State in 2016^32^. On the other hand, the least promising scenario (Scenario 3, assumption of extreme priming) could sequester 2.5 Mt of C per year over the State (9.1 Mt of CO_2_e), or 6% of the 159 Mt CO_2_e attributed to the State in 2016^32^. These numbers indicate the sequestration potential of biochar application on the sugarcane fields of São Paulo. As far as we are aware, there are no long-term field experiments on the C sequestration potential of sugarcane biochar in Brazil or elsewhere, with which to test our model predictions. This needs to be done in future.

We have not allowed for possible increases in sugarcane yield over time with biochar incorporation. The literature reports wide ranges in yield effects of biochar depending on crop type, soil conditions, climate, and biochar characteristics^33–39^. In general, yield responses are smaller for perennial crops, such as sugarcane, compared to annual crops^34,37,38^. In any case, the management of sugarcane plantations in São Paulo is such that yields are already apparently optimized and there is little room left for improvement. According to the IBGE (Instituto Brasileiro de Geografia e Estatística), yields stabilized around 2007^40^.

### Potential additional greenhouse gas emissions

Biochar addition may affect N_2_O emissions during nitrification and denitrification in soils, at least in the first year following application^41,42^. Meta-analyses of field studies indicate 28% ± 16% lower N_2_O emissions with various types of biochar application^43^, but the wide range of results indicate this is one of the most uncertain components of the GHG balance^6^.

Similar analysis suggest biochar may decrease CH_4_ emissions from soils, particularly if flooded or acidic or both^44^. However, biochar applied to unflooded neutral or alkaline soils can increase CH_4_ emissions. Taking landscape diversity and CH_4_ uptake (oxidation) into account, it has also been suggested that biochar does not affect net CH_4_ release^45^.

Other biochar aspects that potentially influence the GHG balance include the potential reduced need for irrigation due to improved soil water holding capacity with biochar^41^, reduced fertilizer requirement due to the phosphorus and potassium available in the applied biochar^41,46^ and reduced nitrogen leaching losses^47^.

While the model provides an efficient way to predict potential increases in SOC stocks in sugarcane fields following biochar addition, this constitutes only part of the GHG balance of the overall practice. Emissions during biochar production, transport and application and those discussed above should be considered in a full Life Cycle Assessment of the integration of biochar into sugarcane systems, so as to provide a more accurate figure for the carbon sequestration potential.

## Methods

### Sugarcane production characteristics

In our scenarios, we have assumed that 7 t DM trash ha^−1^ year^−1^ is left on the field to assist active cycling of organic matter^48–50^. We assumed that trash amounted to 140 kg DM per tonne of harvested cane^2,51^, in agreement with previous work^5^. Likewise, we assumed that the potentially available bagasse (pith and rind from cane progressing) amounted to 140 kg DM per tonne of harvest cane^51,52^. We used these data to determine the input of fresh C to the field for three different scenarios and a baseline (“Modelled Scenarios” section). The calculation draws on the estimated C content for trash and/or bagasse, adding estimates for the C input from root decay, root exudates, plus vinasse and filter cake (the aqueous and suspended solid fractions from the effluent of cane processing). The potential biochar C attributable to each hectare per year was obtained based on the biochar yield and C content of sugarcane trash and bagasse biochar.

We considered the seven soil types covering the sugarcane area of São Paulo and obtained meteorological data from 10 weather stations across and surrounding the area. Details of the soils and meteorological stations, a map of the study area, and details on the data used for soil C stock modelling are given in the Supplementary Table S1 to S4.

### Soil carbon model

**Figure 6:**
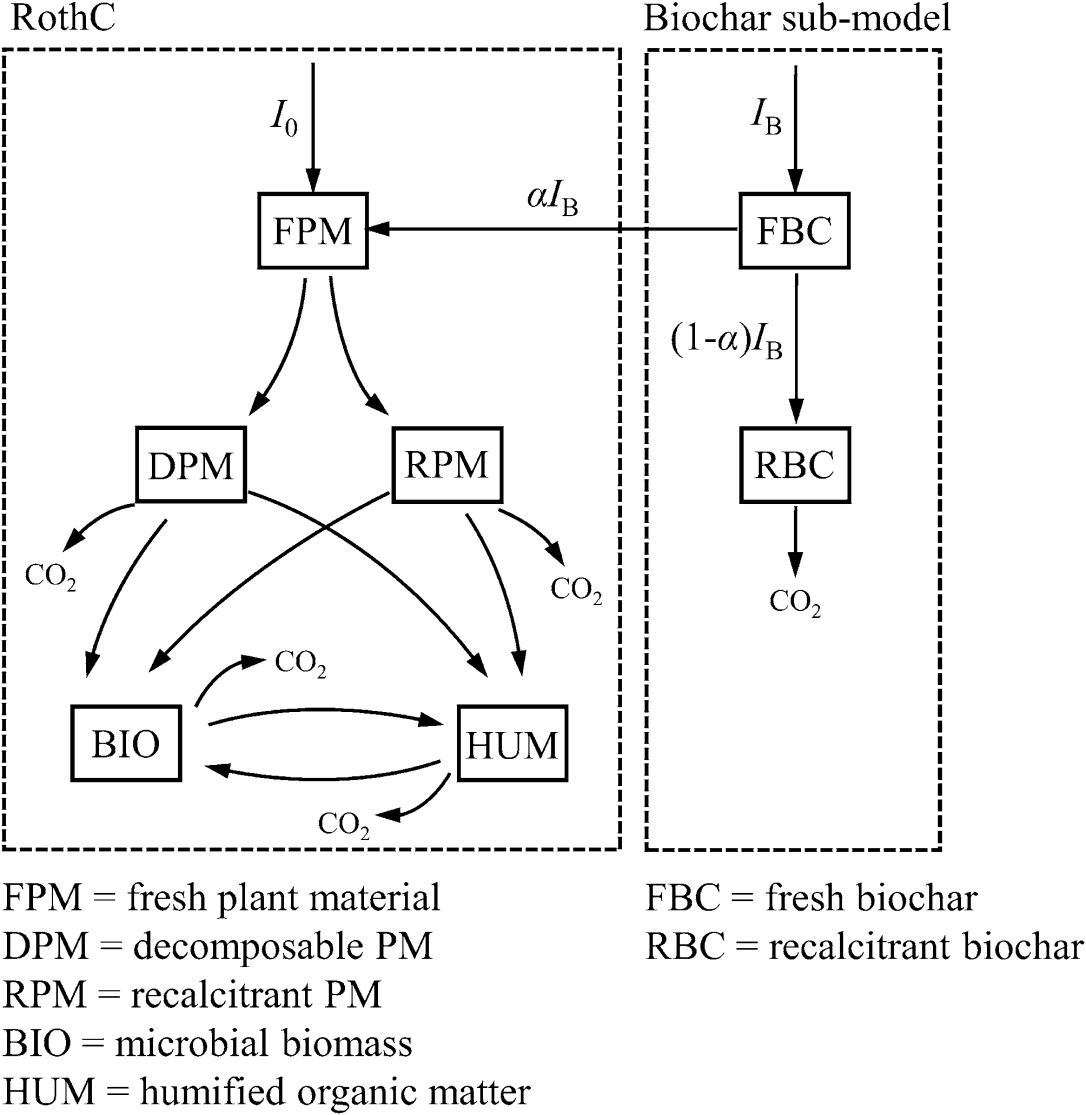
Soil carbon model. RothC is combined with a sub-model for the decomposition of fresh biochar. Details in text.

We combined RothC with a sub-model for biochar decomposition. In RothC, fresh plant material (influx *I*_0_) is divided between two pools of differing decomposability (DPM and RPM) which decompose to two SOC pools of differing decomposability (BIO and HUM), which then inter-convert (Fig. 6)^53^. Each pool decomposes with first order kinetics, and in each inter-pool transformation, a proportion is lost as CO_2_.

In the biochar sub-model, a proportion, *α*, of the C in fresh biochar (influx *I*_B_) is treated as fresh plant material and added to FPM pool in RothC (flux *I*_B_ = *αI*_B_). The remaining, recalcitrant material (RBC) decomposes very slowly releasing CO_2_. The products of such slow decomposition will have a minor impact on soil C pools (BIO ad HUM) and can be neglected for simplicity. Hence

1 $${{{\text{d}}C_{{{\text{RBC}}}} } \mathord{\left/ {\vphantom {{{\text{d}}C_{{{\text{RBC}}}} } {{\text{d}}t}}} \right. \kern-\nulldelimiterspace} {{\text{d}}t}} = \left( {1 - \alpha } \right)I_{{\text{B}}} - k_{{{\text{RBC}}}} C_{{{\text{RBC}}}}$$

where *C*_RBC_ is the concentration of RBC and *k*_RBC_ is its decomposition rate constant.

We nominally parameterised the coupled models for biochar produced by slow pyrolysis of bagasse and trash at 550 °C. Lacking sufficient specific data we considered 3% of the applied biochar C as the portion reflecting the degradability of fresh plant material (DPM/RPM ratio of 1.44, similar to sugarcane residues), based on a recent meta-analysis of all biochar types^13^ and which was consistent with previous assessments^54,55^. Based on a sugarcane biochar specific incubation study, we assumed that the remaining fraction would decompose at a rate of 11.9% over 100 year^18^, i.e. mean residence time = 840 year, consistent with 560 ± 480 year calculated by Wang et al.^13^. Hence *k*_RBC_ = 0.00119 year^−1^. This *k* value is consistent with recent IPCC guidelines for estimating the change in SOC stock in mineral soils from biochar amendment^56^.

We explored the sensitivity of the model to positive priming of SOC turnover by biochar by increasing the RothC rate constants of all pools except RBC by (a) 21% based on Wang et al.’s^13^ meta-analysis, which reported a mean 21% increase in SOC turnover with biochar applications to sandy soils; and (b) by 91% based on increased sucrose turnover with sugarcane bagasse biochar in an incubation experiment with a simulated soil^57^. We consider the latter to indicate the maximum possible positive priming effect of biochar, since most SOC is highly stabilised and unlikely to respond as the labile material. Since published meta-analyses encompass short-term study of large doses and single additions, our permanent increase of C turnover across all pools is highly conservative with respect to SOC.

We used the RothC model from the package ‘SoilR’^58^, coded in R^59^. We amended the SoilR code to obtain output that better corresponded to the original RothC model for its own calibration data (see Supplementary Information). At steady state, before any biochar application, the ‘inert’ C stock for São Paulo soils was calculated according to Falloon et al. (1998)^60^. Model results are presented using ArcGIS 10.5.1.^61^. To smoothen the values between climate stations, we interpolated the meteorological data using inverse distance weighting, which uses the distance of known points to unknown points to estimates their values^62^. The graphical representation of the results were created using R package ‘ggplot2’^63^ from R software version (3.5.1)^59^.

### Modelled scenarios

**Figure 7:**
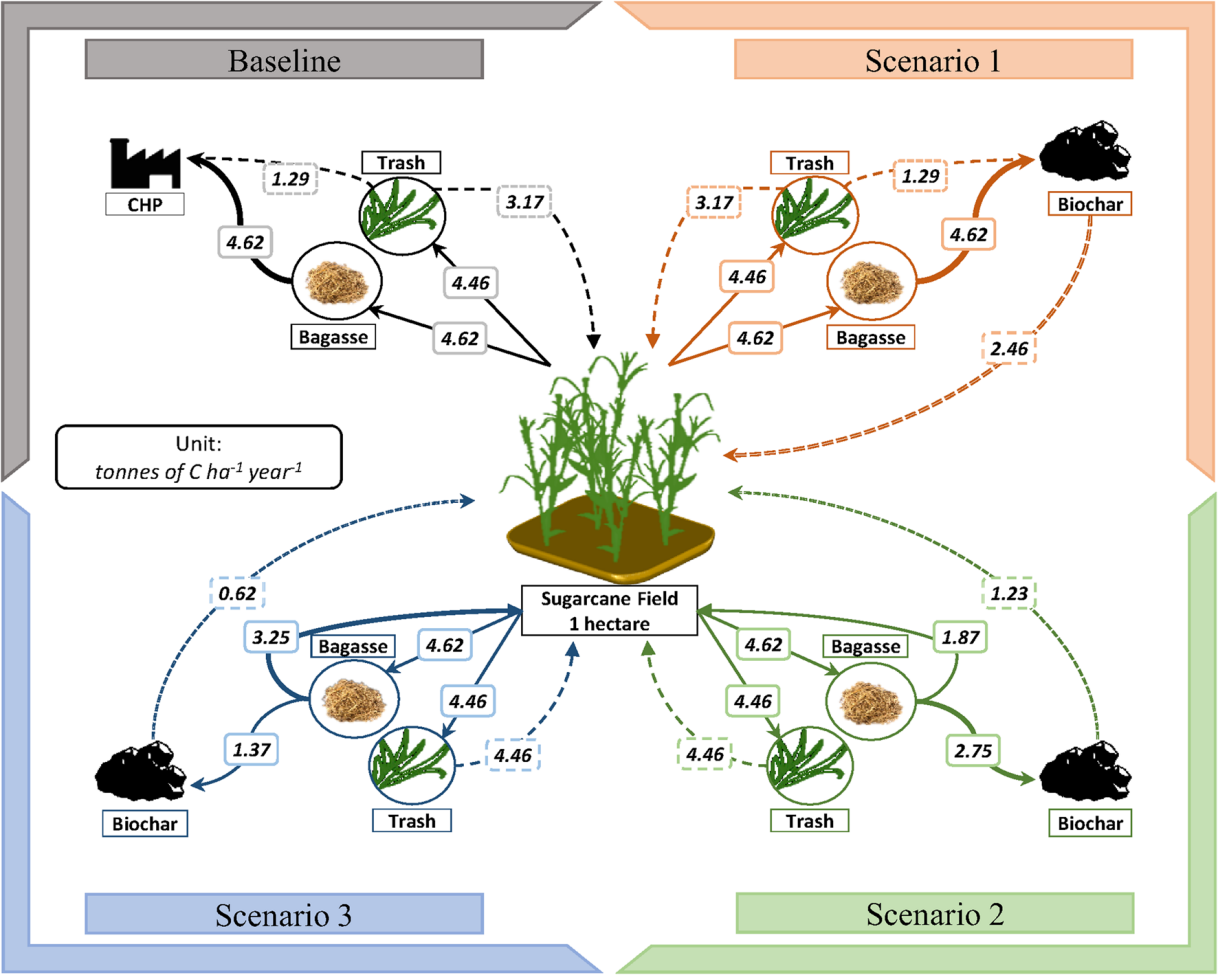
Scenario flowchart representing the fate of the bagasse and trash C produced per hectare per year on a sugarcane field in São Paulo. ‘CHP’ stands for ‘combined heat and power plant’.

We considered three scenarios for biochar and organic matter management together with a baseline (Fig. 7). We used the baseline scenario to obtain the steady-state C stock expected in the long-term under emergent practice and in the absence of climate change. It considers that 67% of sugarcane trash amounting to 3.17 t C ha^−1^ year^−1^ (7 t DM ha^−1^ year^−1^) is left on the field. The remaining trash is supplied to a combined heat and power plant, along with 100% of the bagasse. The total fresh organic C input to the field is 6.57 t C ha^−1^ year^−1^, since the C from trash is supplemented by below ground inputs (estimate for decaying roots and root exudates, after allowing for rhizome growth) as well as the application of 100% vinasse and filter cake (Supplementary Table S4). We ran the model for 500 year to determine the steady-state SOC content, allowing for the effects of intermittent replanting^64^. Scenarios 1, 2 and 3 represent different managements relative to this baseline.

In Scenario 1, 1.29 and 4.62 t C ha^−1^ year^−1^ of the available trash and bagasse respectively (i.e. 100% of each) are supplied to the pyrolysis plant, which yields 2.46 t biochar C ha^−1^ year^−1^ to be returned to the sugarcane fields (calculation details in Supplementary Information). These are repeated annual applications. The other inputs are unchanged. In Scenario 2, only half of the potential biochar production from trash and bagasse is realised (i.e. 1.23 t biochar C ha^−1^ year^−1^—from 60% of the available bagasse). The remainder is left in (or returned to) the field, increasing fresh organic C input from 6.57 in Baseline and Scenario 1 to 9.73 t C ha^−1^ year^−1^. In Scenario 3, the maximum potential biochar production is further diminished to one-quarter of the potential, amounting to 0.62 t biochar C ha^−1^ year^−1^ (from 30% of the available bagasse). The remainder is left in (or returned to) the field, increasing fresh organic C input to 11.1 t C ha^−1^ year^−1^.

All the scenarios include a loss of 5% of the trash dry matter during the stalk collection and transport^65^. Scenarios 1, 2 and 3 are run for 5, 10, and 20 year, starting at the steady-state (baseline) C stock. A maximum of 20 year was chosen to match the higher experimental biochar additions tested in any cropping system^34,66^.

### Model evaluation

To attach confidence to our analysis, we decided to test the biochar sub-model that we coupled to RothC. The established RothC model has successfully described SOC turnover in many parts of the world including Brazil^67,68^. The relevance of the coupled biochar sub-model is not diminished relative to RothC, as it contains parameters derived from meta-analysis and reflecting all published biochar research, so although it would have been ideal to calibrate against sugarcane crop data, it was not essential. Since there are to our knowledge no published data on long-term field experiments on biochar in sugarcane, we instead used data from a wheat–maize study in China^24^. In this study 0, 1.16, 3.48 or 5.79 t of rice straw biochar C ha^−1^ were applied to both the wheat and following rice crop in each year. This study is useful as it is both long-term and involves repeated additions of biochar to all land, representative of our modelled scenarios. Data required for the model evaluation are reported in the Supplementary Information (Supplementary Table S5).

The biochar literature suggests that some biochar is transported after topsoil application to sub-soil layers by rainfall (infiltration) and/or bioturbation^69,70^. Between 0.5 and 14.75% of applied biochar is found below the application depth (ranging between 5 and 20 cm) after one year^69–73^, depending on the biochar particle size^69^, rainfall amount^69^ and soil texture and porosity^69,70^. Biochar movement to subsoil layers or beyond will not reduce net C sequestration and may even increase it compared with more-transient storage in the topsoil^74,75^, but could introduce errors in field experiments with limited depth of sampling. For the purpose of model evaluation by deriving a relation between percentage downward loss and soil texture using data reported in the literature^69–73^, a probable loss could be derived for sandy loam soil of the wheat–maize experiment used in calibration data^24^. The value was 5.18% of the applied biochar per year.

## Supplementary information

Supplementary Information 1.

## Data Availability

All data generated or analysed during this study are included in the published article and Supplementary information.
