# Supplementary material for: Modelling the potential for soil carbon sequestration using biochar from sugarcane residues in Brazil
Source: Sci Rep. 2020 Nov 10;10:19479. doi: 10.1038/s41598-020-76470-y (PMC7655943; doi:10.1038/s41598-020-76470-y)
Supplement: Supplementary file 1 — Supplementary Information 1. [file 41598_2020_76470_MOESM1_ESM.docx]

Supplementary information

**Modelling the potential for soil carbon sequestration using biochar from sugarcane residues in Brazil**

David Lefebvre ^1,*^, Adrian Williams ^1^, Jeroen Meersmans ^1, 2^, Guy J.D. Kirk ^1^, Saran Sohi ^3,^ Pietro Goglio ^1, 4^, and Pete Smith ^5^

^1^ School of Water, Energy and Environment, Cranfield University, College Road, Bedford, MK43 0AL, UK

^2^ TERRA Teaching and Research Centre, Gembloux Agro-Bio Tech, University of Liège, Gembloux 5030, Belgium

^3^ UK Biochar Research Centre (UKBRC), School of GeoSciences, Crew Building, University of Edinburgh, EH9 3FF, United Kingdom

^4^ Wageningen Economic Research, Wageningen University & Research, Leeuwenborch, Hollandsweg 1, 6706KN Wageningen, The Netherlands

^5^ Institute of Biological and Environmental Sciences, University of Aberdeen, 23 St Machar Drive, Aberdeen AB24 3UU, UK

Contents

[Meteorological and soil parameters S3](#_Toc35596641)

[Calculation – Biochar production (example for Scenario 1) S8](#_Toc35596642)

[Changes to RothC model from package ‘SoilR’ coded in R S9](#_Toc35596643)

[Model value outputs and literature data comparison S10](#_Toc35596644)

[Evaluation of the Model S11](#_Toc35596645)

[Evaluation Dataset S11](#_Toc35596646)

[Additional Evaluation work S12](#_Toc35596647)

[Relevance of Incubation Studies S15](#_Toc35596648)

# Meteorological and soil parameters

Characteristics of the soil types covering Sao Paulo state’s sugarcane area were obtained from the Harmonized World Soil Database ^1^ (Table S1).

**Table S1.** Main characteristics of the soil type selected as given by the harmonized world soil database ^1^.

| Parameter | Soil 1 | Soil 2 | Soil 3 | Soil 4 | Soil 5 | Soil 6 | Soil 7 |
| --- | --- | --- | --- | --- | --- | --- | --- |
| Soil Unit Symbol (FAO 90) | FRr | LXh | FRr | FRh | ACf | ARo | ACf |
| Soil Unit Name (FAO 90) | Rhodic  Ferralsols | Haplic  Lixisoil | Rhodic  Ferralsols | Haplic  Ferralsols | Ferric  Acrisols | Ferralic  Arenosols | Ferric  Acrisols |
| Top Soil Texture | Fine | Coarse | Coarse | Fine | Coarse | Coarse | Medium |
| Topsoil Sand Fraction (%) | 13 | 91 | 84 | 52 | 88 | 92 | 67 |
| Topsoil Silt Fraction (%) | 25 | 3 | 4 | 7 | 2 | 3 | 7 |
| Topsoil Clay Fraction (%) | 62 | 6 | 12 | 41 | 10 | 5 | 26 |
| Topsoil USDA Texture Classification | Clay  (heavy) | Sand | Loamy  sand | Sandy  clay | Loamy  sand | Sand | Sandy  clay loam |
| Topsoil Bulk Density (kg/dm3) | 1.20 | 1.49 | 1.44 | 1.45 | 1.52 | 1.52 | 1.40 |
| Topsoil Organic Carbon (% weight) | 1.20 | 0.30 | 0.67 | 1.90 | 0.60 | 0.84 | 0.97 |

Topsoil refers to the 0 – 30 cm depth layer

Latitude, temperature and precipitation data from 10 meteorological stations representing Sao Paulo state sugarcane area were obtained from the CLIMWAT database ^2^ (Table S2).

**Table S2.** Latitude, longitude, yearly average temperature and monthly precipitation values of the different meteorological stations considered from ClimWat database ^2^.

| Meteorological  Station | Latitude  (degree) | Longitude  (degree) | Average  Temperature  (°C) | Average  Precipitation  (mm/month) | Average  Evapotranspiration  (mm/month)* |
| --- | --- | --- | --- | --- | --- |
| Pocos de Caldas | -21.85 | -46.56 | 18.3 | 141.5 | 93.0 |
| Franca | -20.55 | -47.43 | 19.1 | 135.3 | 95.4 |
| Frutal | -20.03 | -48.93 | 24.0 | 127.5 | 140.9 |
| Tres Lagoas | -20.78 | -51.63 | 24.6 | 108.7 | 151.8 |
| Ribeirao Preto | -21.18 | -47.71 | 20.4 | 128.8 | 104.9 |
| Piracicaba | -22.71 | -47.63 | 20.7 | 103.4 | 108.3 |
| Bauru | -22.31 | -49.06 | 20.7 | 121.4 | 108.2 |
| Avare | -23.1 | -48.91 | 20.8 | 110.2 | 108.6 |
| Londrina | -23.38 | -51.18 | 21.3 | 135.2 | 113.4 |
| Campinas | -22.9 | -47.08 | 19.7 | 116.2 | 100.9 |
| *Thornthwaite potential evapotranspiration was calculated using the R software package “SPEI” ^3^and divided by 0.75, as suggested by the RothC user guide ^4^. | | | | | |

Out of 70 potential soil type/meteorological station combination, only 36 actually represented in Sao Paulo State (Table S3).

**Table S3.** The actual existing soil type/meteorological station combinations present in the state of Sao Paulo.

|  | **Soil 1** | **Soil 2** | **Soil 3** | **Soil 4** | **Soil 5** | **Soil 6** | **Soil 7** |
| --- | --- | --- | --- | --- | --- | --- | --- |
| Tres Lagoas | X | X | X |  |  |  |  |
| Pocos de Caldas | X |  |  | X | X |  |  |
| Ribeirao Preto | X | X | X | X | X |  |  |
| Piracicaba |  |  | X | X | X | X |  |
| Londrina | X | X | X |  |  |  |  |
| Frutal | X | X | X |  |  |  |  |
| Franca | X |  |  |  | X |  |  |
| Campinas |  |  | X | X | X |  |  |
| Bauru | X | X | X | X |  | X |  |
| Avare | X |  | X |  | X | X | X |

The spatial distribution of the different soil types and sugar cane fields as well as the locations of the meteorological stations are shown in Fig. S1


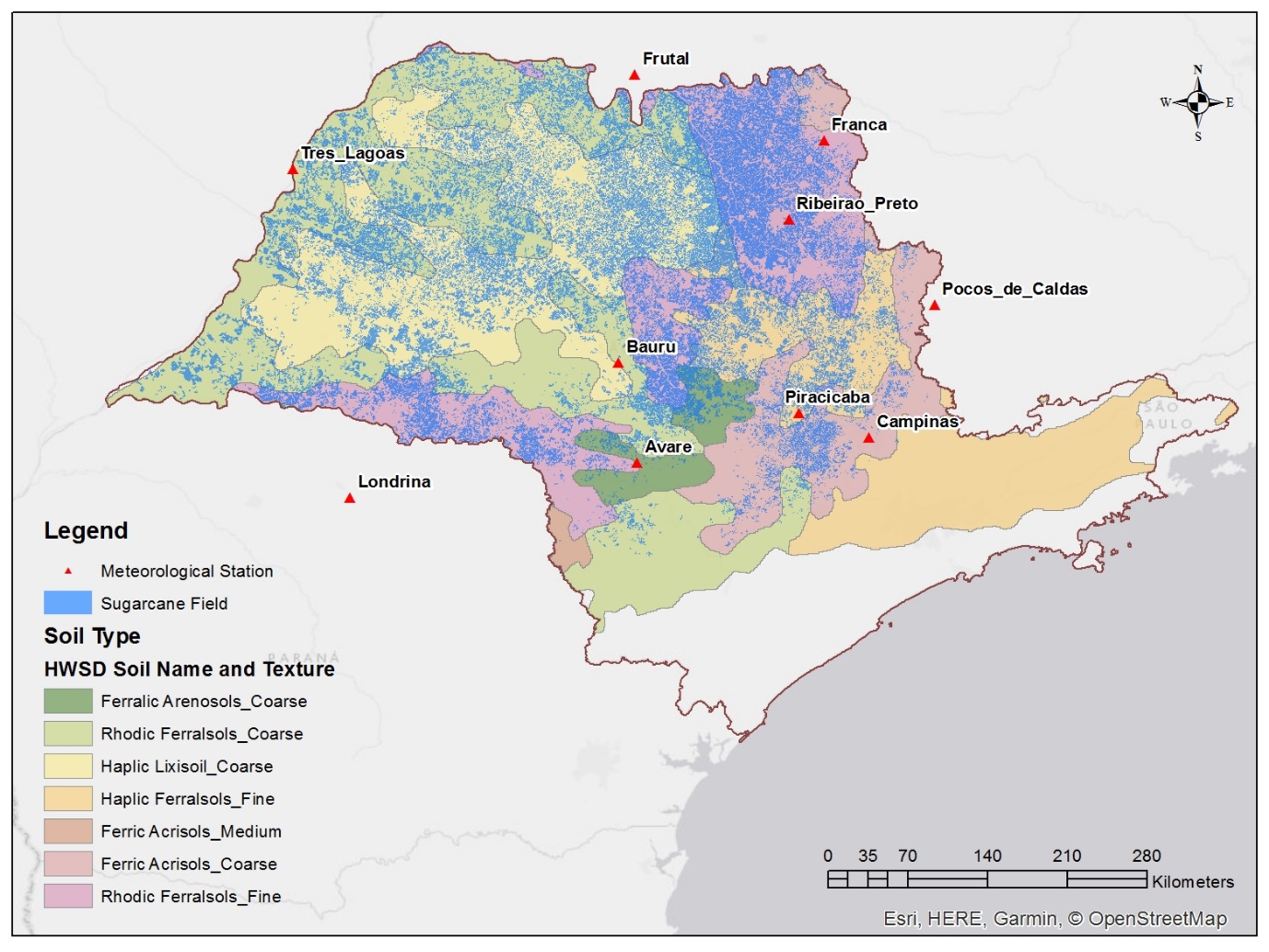


**Figure S1.** The study area (State of Sao Paulo – Brazil) with; (i) the 7 different soil types according to the Harmonized World Soil Database ^1^, (ii) the location of the meteorological stations considered in the study ^2^ and (iii) the sugarcane fields according to the CANASAT project ^5^. Map generated using ArcGIS 10.5.1 ^6^.

**Table S4.** Parameter settings for soil carbon and biochar production calculations.

| Parameter | Value | Unit | Reference |
| --- | --- | --- | --- |
| Area in Sugarcane in ha (2014 - all form) | 5,768,172 | ha | ^5^ |
| Estimated sugarcane yield in Sao Paulo (2018-19) | 74,037 | TC* ha^-1^ | ^7^ |
| Filter Cake yearly addition | 0.733 | tonnes ha^-1^ | ^8^ |
| Vinasse yearly addition | 31 | m^3^ ha^-1^ | ^8^ |
| Filter Cake Carbon content | 32.5 | % | ^9^ |
| Vinasse carbon content | 1.99 | kg m^3^ | ^10^ |
| Average Bagasse production (dm) | 140 | kg dm TC^-1^* | ^11,12^ |
| Bagasse Carbon content | 44.6 | % | ^13^ |
| Average trash per tonne of cane (dm) | 140 | kg dm TC^-1^* | ^12,13^ |
| Trash losses in sugarcane harvest | 5 | % TC* | ^14^ |
| Trash Carbon content | 45.27 | % | ^13,15^ |
| Amount of trash to be left on field | 7 | tonnes dm ha^-1^ | ^13,16–19^ |
| Yearly carbon input from sugarcane root system | 1.88 | tonnes ha^-1^ | ^19^ |
| Yearly carbon input from root exudates | 65 | % of root C content | ^20^ |
| Sugarcane trash biochar (550°C) C content (% dm) | 41.01 | % | ^21^ |
| Sugarcane bagasse biochar (550°C) C content (% dm) | 63.78 | % | ^21^ |
| Bagasse Biochar production Yield (550°C) | 31.3 | % | ^22^ |
| Trash Biochar production Yield (550°C) | 33.6 | % | ^22^ |
| Biochar weight considered labile | 3 | % | ^23^ |
| Biochar weight considered recalcitrant | 97 | % | ^23^ |
| Sugarcane biochar C lost after 100 years (525°C) | 11.9 | % | ^24^ |
| Soil thickness for modelling activities | 30 | cm | ^1^ |
| DPM:RPM ratio for modelling activities | 1.44 | - | ^4^ |
| * TC = tonne of cane | | |  |

# Calculation – Biochar production (example for Scenario 1)

In Scenario 1, 1.29 and 4.62 t C ha^-1^ yr^-1^ of the available trash and bagasse respectively (i.e. 100% of each) are supplied to the pyrolysis plant. These figures correspond to 2.85 t and 10.365 t dm of trash and bagasse, respectively, ha^-1^ yr^-1^ (see carbon content of trash and bagasse Table S4).

From there, the equation is:

$$TotalBCC=Wt\left( dm \right)*tBCY*tBCCc+Wb\left( dm \right)*bBCY*bBCCc$$

With:

TotalBCC: total biochar carbon applied [t biochar C ha^-1^ yr^-1^]

Wt(dm): trash dry weight available [t ha^-1^ yr^-1^]

tBCY: trash biochar production yield [%]

tBCCc: trash biochar carbon content [%]

Wb(dm): bagasse dry weight available [t ha^-1^ yr^-1^]

bBCY: bagasse biochar production yield [%]

bBCCc: bagasse biochar carbon content [%]

Using values for scenario 1 and from the Table S4 hereabove:

$$TotalBCC=2.85*0.336*0.4101+10.365*0.313*0.6378$$

TotalBCC equals to 2.46 t biochar C ha^-1^ yr^-1^.

# Changes to RothC model from package ‘SoilR’ coded in R

RothC soil carbon model from the package ‘SoilR’ ^25^ in R ^26^ (hereafter referred as ‘RothC_R’) was amended to correspond better with the output values obtained through the RothC original Windows version. From the original R script we:

1. We added a ‘Soil Cover Factor’, present in the original RothC, which modifies the rate constants and takes the monthly value of 0.6 if the soil is vegetated or 1 if it is bare, with an averaged value considering 9 months covered and 3 months bare for sugarcane ^27^.
2. We modified the Top Soil Moisture Deficit (‘fW rate factor modifier’) which, originally, takes the monthly value of 1 if the soil is vegetated or 1.8 if it is bare ^4^ with an averaged value based on the same covered and bare ratio than for the ‘Soil Cover Factor’.
3. We multiplied the Soil Cover Factor (SCF) by the rate modifier for temperature (fT) and the updated rate modifier for soil moisture (fW), so the final modifier (Xi) takes into account the temperature, moisture and soil cover.

## Model value outputs and literature data comparison

The steady state carbon stock values produced by the Windows and R versions of RothC were highly similar (Figure S2). The slight observed differences may be due to the use of yearly averaged values for the ‘Topsoil Moisture Deficit’ coverage modifying factor and the ‘Soil Cover Factor’ (discussed here above) in the RothC_R whereas in Windows version monthly values where used. Our overall mean value of soil carbon stock at steady state (represented by the purple line in Figure S2) is consistent with the mean top 30 cm SOC stock values for Sao Paulo State as reported in the literature ^19,28,29^ (see black boxplot in figure S2).


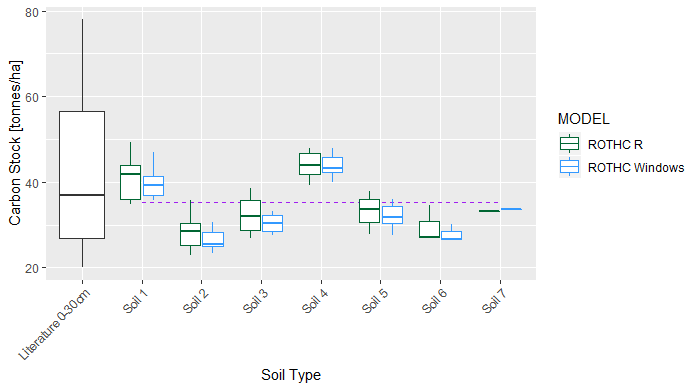


**Figure S2.** Soil carbon stock values at steady state obtained through the R (green) and the Windows (blue) version of RothC. The boxplot combines the values obtained for the different meteorological stations relevant to each soil type. The black boxplot represent the spread of the soil carbon stock under sugarcane cultivation in Sao Paulo found in the literature ^19,28,29^. The single line for Soil 7 is due to the only meteorological station attributed to this soil type (see Table S3). The purple dotted line represents the averaged value when all relevant soils and climate station combinations are considered. The end of the upper (lower) whisker represent the largest (smallest) value within 1.5 times interquartile range above (below) 75th (25th) percentile, as defined by the ggplot2 package of R software version (3.5.1) ^26^.

# Evaluation of the Model

## Evaluation Dataset

Evaluation of our model was made using the data from Liu et al. ^30^ who measured soil carbon stock increase of a sandy loam soil in China under yearly rotations of wheat-maize, in which both were grown to harvest. The measurement was made after 5 years (10 crop seasons) while biochar application was made 10 times in total (once after each harvest). Data needed for the evaluation of our model are reported in Table S5.

**Table S5**: Evaluation dataset

| Parameter | Value | Unit | Reference |
| --- | --- | --- | --- |
| Cultivation Period | October to June (wheat) and June to September for maize | - | ^30^ |
| Biochar application rate | 0; 2.25; 6.75; 11.35 | tonnes ha^-1^ twice a year | ^30^ |
| Biochar feedstock | Rice straw (500°C) | - | ^30^ |
| Biochar application depth | 15 | cm | ^30^ |
| Biochar carbon content | 51.5 | % | ^30^ |
| Biochar recalcitrant pool mean residence time | 840 ^‡^ | Years | ^24^ |
| Biochar labile content | 3 ^‡^ | % | ^31,32^ |
| Soil clay content | 22.6 | % | ^30^ |
| Soil sand content | 46.3 | % | ^30^ |
| Site latitude | 35.0 N | Degree | ^30^ |
| Mean annual temperature | 13.9 | °C | ^30^ |
| Mean annual rainfall | 605 | mm | ^30^ |
| Average soil carbon stock at steady state | 16.65 | tonnes C ha^-1^ | ^30^ |
| Carbon content measurement depth | 15 | cm | ^30^ |
| Fresh yearly carbon input* | 0.99 | tonnes C ha^-1^ | ^4^ |
| DPM:RPM ratio | 1.44 | - | ^4^ |
| * Calculated using climate, soil, and management data in RothC.  ‡ Considered as identical to our assessment using sugarcane biochar. | | | |

## Additional Evaluation work

Long-term experiments reporting soil carbon stock changes after biochar addition are scarce. In addition to the first evaluation dataset from Liu et al. ^30^ (shown in the manuscript), a second attempt to evaluate our model was made using the data from Rogovska et al. ^33^ who measured soil carbon content and bulk density of a Mollisol in Iowa (US) under maize cultivation two years after biochar application. The same data was used by Archontoulis et al. ^34^ to validate their biochar sub-model for the Agricultural Production System Simulator (APSIM).

Parameter settings for the additional evaluation of our model are reported in Table S6.

**Table S6:** Additional evaluation dataset

| Parameter | Value | Unit | Reference |
| --- | --- | --- | --- |
| Maize cultivation period | May to October | - | ^33^ |
| Biochar application rate | 0; 19; 38; 58; 77; 96 | tonnes ha^-1^ | ^33^ |
| Biochar feedstock | Mixed hardwood | - | ^33^ |
| Majority of biochar particle size | < 1 | mm | ^33^ |
| Biochar application depth | 30 | cm | ^33^ |
| Biochar carbon content | 78 | % | ^33^ |
| Biochar lost during application | 2 | % | ^34^ |
| Biochar recalcitrant pool mean residence time | 500 | Years | ^34^ |
| Biochar labile content | 13 | % | ^34^ |
| Soil clay content | 21 | % | ^34^ |
| Soil sand content | 41.6 | % | ^34^ |
| Soil organic carbon content | 2.03 | % | ^34^ |
| Site latitude | 42.0 | Degree | ^34^ |
| Mean annual temperature | 9.3 | °C | ^34^ |
| Mean annual rainfall | 900 | mm | ^34^ |
| Average soil carbon stock at steady state | 33.6 | tonnes C ha^-1^ | ^33^ |
| Carbon content measurement depth | 15 | cm | ^33^ |
| Fresh yearly carbon input* | 2.554 | tonnes C ha^-1^ | ^4^ |
| DPM:RPM ratio | 1.44 | - | ^4^ |
| Additional fresh C input at the beginning of the experiment‡ | 9.1 | tonnes C ha^-1^ | ^33^ |
| * Calculated using climate, soil, and management data in RothC.  ‡ The experiment includes additional maize straw input on the field after biochar application to avoid loss by erosion. | | | |

Comparisons between simulated and measured values (Figure S3) shows good agreement up to the application rate of 38 t of biochar ha^-1^. However, the difference between simulated and measured soil carbon stock is large for the application rates of 58, 77, and 96 t of biochar ha^-1^. Similar results were obtained by Archontoulis et al. ^34^ during their model validation attempt of their APSIM biochar sub-model.


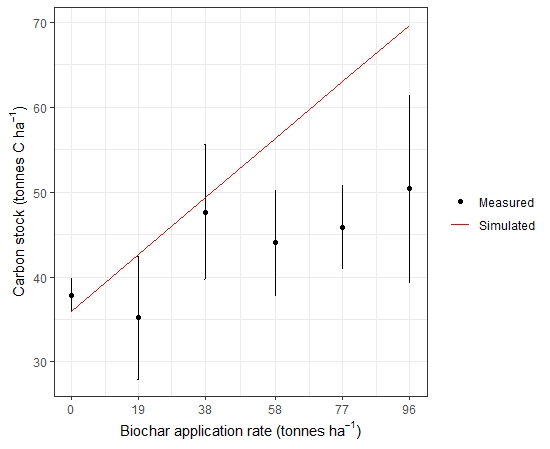


**Figure S3:** Comparison between measured ^33^ (black dots ± SD) and simulated (red line) values using our modified RothC model. The soil carbon measured values originates from Rogovska et al. ^33^ two years after biochar application.

The sudden decrease in measured soil carbon stock from 58 t of biochar ha^-1^ onward translates a substantial loss of carbon. This loss is not expected to be due to an increase in mineralization rate of the biochar with increasing application rate (the opposite should occur, according to a recent review ^35^). Hence, this particular loss of biochar must be the consequence of factors, which are not taken into consideration by the RothC carbon model.

The literature suggests that biochar could be transported vertically through the soil profile towards deeper soil layers due to rainfall and/or bioturbation ^36,37^, effectively making it absent from the topsoil carbon measurements. The share of biochar found under the application depth after one year ranges from 0.5% to 14.75% ^36–40^. The main factors influencing the vertical migration of biochar particle are thought to be the biochar particle size ^36^, the soil texture and porosity ^36,37^, and the rainfall amount ^36^. We interpolated the potential amount of biochar that could have been lost to deeper soil layers during the experiment of Rogovska et al. ^33^ from the percent loss reported in the literature based on the sand content of the soils studied. The loss of biochar from leaching in Rogovska et al. ^33^ was estimated at 5.9% of the applied biochar per year. This value is around half the values reported by Obia et al. ^36^ for similar rainfall amounts (≈1000 mm year^-1^) and biochar particle size (≤ 1 mm) but higher sand content. Accounting for this loss brings our simulated values closer to the measurements (Figure S4).

Lateral transportation (erosion) of biochar is also expected to be an important pathway of biochar in soils ^31^. Recent studies suggest that 20% to 40% of the biochar could be lost by erosion a year or two after its application, even when the biochar is mixed in the first 10 cm of soil ^36,40^. In Rogovska et al. ^33^, the additional fresh organic matter input after biochar application aimed at reducing this potential erosion. No additional organic matter was reported for the second year of the experiment, which may have led to the loss of some biochar particles, however, the erosion of biochar is expected to decrease with time, as biochar particles are incorporated into aggregates ^31^. The lack of data prevents any precise assessment of biochar loss by erosion. Staying in the range of the number reported in the literature, we considered that an approximated 15% of the biochar applied was lost to erosion two years after its application (Figure S4).


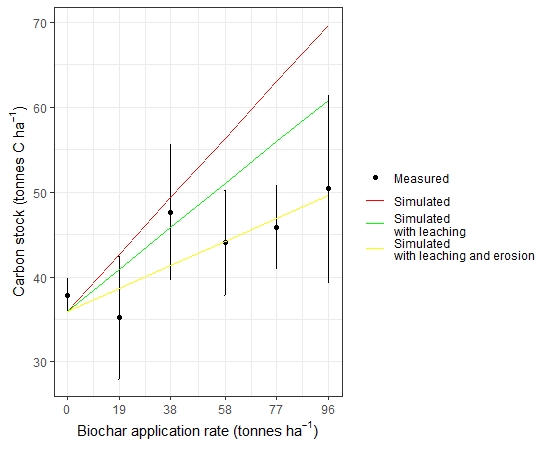


**Figure S4:** Carbon stock (t ha^-1^) 2 yr after biochar addition. Comparison between measured values ^33^ (black dots ± SD), simulated (red line) values using our modified RothC model, simulated values accounting for potential leaching of biochar particles (green line), and simulated values accounting for potential downward (leaching) and lateral migration (erosion) of biochar particles (yellow line).

The loss of biochar through percolation and erosion, when reported, usually exceeds the loss through mineralization and is a known parameter causing underestimation of persistence of biochar in soils in field studies ^39,41^.

The reported, although scarcely documented, loss of biochar from its application layer either by leaching or erosion may render the evaluation of biochar models tedious. However, it does not impede its carbon sequestration potential as biochar in subsoil layers or deposited in aquatic environments may be more stable than when stored in the topsoil ^42,43^.

## Relevance of Incubation Studies

Incubation studies offer another type of measurement where biochar mineralization rate is precisely determined while avoiding other type of losses. However, they are often criticized for not representing the soil-plant system realistically enough ^23^. Regarding biochar mineralization, critics argue that the optimal decomposition condition and usually short-term experimental duration lead to higher biochar decomposition rates than in field conditions ^23,44^, while others expect an underestimation of the mineralization rate in incubation studies due to the impossibility of representing the complexity of real-world conditions at small scale ^41^. Figure S5 compares our modelled mineralization rate with values from the literature.


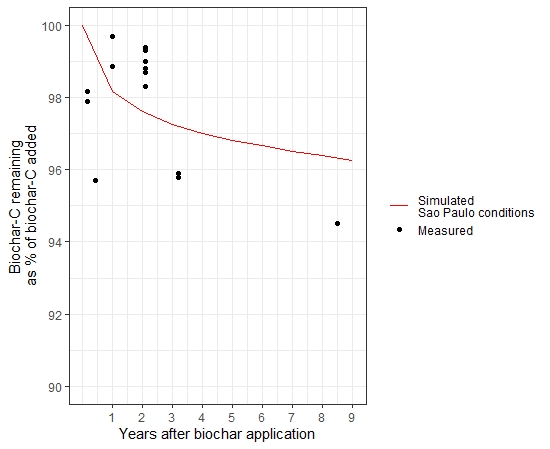


**Figure S5**: Biochar-C remaining as percent of biochar-C added over time. Our simulated values (red line) are based on meta-analysis values ^23^ applied on Sao Paulo conditions. The black dots are data points collected from literature ^44–48^ studying biochar mineralization in various condition through incubation experiments. The model reflects the general trend of these disparate experiments, which show, not-surprisingly, substantial scatter. Without longer term incubation experimental data, we cannot conclude if the model shows systematic bias nor do the experimental data contradict the model’s predictions.

The main factors affecting biochar mineralization rate in incubation studies are the incubation time, the soil characteristics, and the biochar properties ^35^. The results gathered (black dots - Figure S5) originates from studies where biochar was made at a temperature between 400°C and 600°C ^44–48^, which is representative of our simulated 550°C biochar.

REFERENCES

1. FAO. Harmonized world soil database v1.2 | FAO SOILS PORTAL. *Food and Agriculture Organization of the United Nations* www.fao.org/soils-portal/soil-survey/soil-maps-and-databases/harmonized-world-soil-database-v12/en/ (2012).

2. FAO. CLIMWAT 2.0 for CROPWAT. (2006).

3. Beguería, S. & Vicente-Serrano, S. M. SPEI: Calculation of the Standardised Precipitation-Evapotranspiration Index. (2017).

4. Coleman, K. & Jenkinson, D. S. *RothC - A model for the turnover of carbon in soil Model - Model description and users guide*. https://www.rothamsted.ac.uk/sites/default/files/RothC_guide_WIN.pdf (2014).

5. Theodor Rudorff, B. F. Canasat - INPE. http://www.dsr.inpe.br/laf/canasat/tabelas.html (2014).

6. ESRI. ArcGIS Desktop. http://desktop.arcgis.com (2011).

7. CONAB. *Acompanhamento da Safra Brasileira - Cana-de-açúcar - Terceiro levantamento | Dezembro 2018*. https://www.conab.gov.br/info-agro/safras/cana (2018).

8. Cardoso, T. F. *et al.* A regional approach to determine economic, environmental and social impacts of different sugarcane production systems in Brazil. *Biomass and Bioenergy* **120**, 9–20 (2019).

9. George, P. A. O., Eras, J. J. C., Gutierrez, A. S., Hens, L. & Vandecasteele, C. Residue from sugarcane juice filtration (filter cake): Energy use at the sugar factory. *Waste and Biomass Valorization* **1**, 407–413 (2010).

10. de Oliveira, B. G., Carvalho, J. L. N., Cerri, C. E. P., Cerri, C. C. & Feigl, B. J. Soil greenhouse gas fluxes from vinasse application in Brazilian sugarcane areas. *Geoderma* **200**–**201**, 77–84 (2013).

11. Khatiwada, D., Leduc, S., Silveira, S. & McCallum, I. Optimizing ethanol and bioelectricity production in sugarcane biorefineries in Brazil. *Renew. Energy* **85**, 371–386 (2016).

12. Waldheim, L., Monis, M. & Verde Leal, M. R. Biomass Power Generation: Sugar Cane Bagasse and Trash. in *Progress in Thermochemical Biomass Conversion* (ed. Bridgwater, A. .) 509–523 (Blackwell Science Ltd, 2001).

13. Hassuani, S. J., Leal, M. R. L. V. & Macedo, I. de C. *Biomass Power Generation - Sugar cane bagasse and trash*. (PNUD - Programa das Nações Unidas para o Desenvolvimento And CTC - Centro de Tecnologia Canavieira, 2005).

14. Sampaio, I. L. M. *et al.* Electricity Production from Sugarcane Straw Recovered Through Bale System: Assessment of Retrofit Projects. *BioEnergy Res.* **12**, 865–877 (2019).

15. Galdos, M. V., Van Antwerpen, R., Cerri, C. E. P., Paustian, K. & Cerri, C. C. Simulation of Soil Carbon Dynamics under Sugarcane with the CENTURY Model. *Soil Sci. Soc. Am. J.* **73**, 802 (2009).

16. Vasconcelos, A. L. S. *et al.* Greenhouse gas emission responses to sugarcane straw removal. *Biomass and Bioenergy* **113**, 15–21 (2018).

17. Popin, G. V. *et al.* Sugarcane straw management for bioenergy: effects of global warming on greenhouse gas emissions and soil carbon storage. *Mitig. Adapt. Strateg. Glob. Chang.* **25**, 559–577 (2020).

18. Silva, A. G. B., Lisboa, I. P., Cherubin, M. R. & Cerri, C. E. P. How Much Sugarcane Straw is Needed for Covering the Soil? *BioEnergy Res.* **12**, 858–864 (2019).

19. Carvalho, J. L. N., Hudiburg, T. W., Franco, H. C. J. & DeLucia, E. H. Contribution of above- and belowground bioenergy crop residues to soil carbon. *GCB Bioenergy* **9**, 1333–1343 (2017).

20. Bolinder, M. A., Janzen, H. H., Gregorich, E. G., Angers, D. A. & VandenBygaart, A. J. An approach for estimating net primary productivity and annual carbon inputs to soil for common agricultural crops in Canada. *Agric. Ecosyst. Environ.* **118**, 29–42 (2007).

21. Cross, A. & Sohi, S. P. A method for screening the relative long-term stability of biochar. *GCB Bioenergy* **5**, 215–220 (2013).

22. Quirk, R. G. *et al.* Utilization of Biochar in Sugarcane and Sugar-Industry Management. *Sugar Tech* **14**, 321–326 (2012).

23. Wang, J. *et al.* Biochar stability in soil: Meta-analysis of decomposition and priming effects. *GCB Bioenergy* **8**, 512–523 (2016).

24. Zimmerman, A. R. Abiotic and Microbial Oxidation of Laboratory-Produced Black Carbon ( Biochar ). *Environ. Sci. Technol.* **44**, 1295–1301 (2010).

25. Sierra, C. A. & Mueller, M. Package ‘ SoilR ’ - Models of Soil Organic Matter Decomposition. 177 (2015).

26. Core Team, R. R: A language and environment for statistical computing. R Foundation for Statistical Computing. https://www.r-project.org/ (2018).

27. Scarpare, F. V. *et al.* Sugarcane land use and water resources assessment in the expansion area in Brazil. *J. Clean. Prod.* **133**, 1318–1327 (2016).

28. Maia, S. M. F. *et al.* Payback time for soil carbon and sugar-cane ethanol. *Nat. Clim. Chang.* **4**, 605–609 (2014).

29. De Oliveira Bordonal, R. *et al.* Greenhouse gas mitigation potential from green harvested sugarcane scenarios in São Paulo State, Brazil. *Biomass and Bioenergy* **59**, 195–207 (2013).

30. Liu, Y. *et al.* Successive straw biochar amendments reduce nitrous oxide emissions but do not improve the net ecosystem economic benefit in an alkaline sandy loam under a wheat–maize cropping system. *L. Degrad. Dev.* **31**, 868–883 (2020).

31. Foereid, B., Lehmann, J. & Major, J. Modeling black carbon degradation and movement in soil. *Plant Soil* **345**, 223–236 (2011).

32. Woolf, D. & Lehmann, J. Modelling the long-term response to positive and negative priming of soil organic carbon by black carbon. *Biogeochemistry* **111**, 83–95 (2012).

33. Rogovska, N., Laird, D. A., Rathke, S. J. & Karlen, D. L. Biochar impact on Midwestern Mollisols and maize nutrient availability. *Geoderma* **230**–**231**, 340–347 (2014).

34. Archontoulis, S. V. *et al.* A model for mechanistic and system assessments of biochar effects on soils and crops and trade-offs. *GCB Bioenergy* **8**, 1028–1045 (2016).

35. Chao, L., Zhang, W. D. & Wang, S. L. Understanding the dominant controls on biochar decomposition using boosted regression trees. *Eur. J. Soil Sci.* **69**, 512–520 (2018).

36. Obia, A., Børresen, T., Martinsen, V., Cornelissen, G. & Mulder, J. Vertical and lateral transport of biochar in light-textured tropical soils. *Soil Tillage Res.* **165**, 34–40 (2016).

37. Haefele, S. M. *et al.* Effects and fate of biochar from rice residues in rice-based systems. *F. Crop. Res.* **121**, 430–440 (2011).

38. Ventura, M. *et al.* Biochar mineralization and priming effect in a poplar short rotation coppice from a 3-year field experiment. *Biol. Fertil. Soils* **55**, 67–78 (2019).

39. Singh, B. P. *et al.* In situ persistence and migration of biochar carbon and its impact on native carbon emission in contrasting soils under managed temperate pastures. *PLoS One* **10**, 1–20 (2015).

40. Major, J., Lehmann, J., Rondon, M. & Goodale, C. Fate of soil-applied black carbon: Downward migration, leaching and soil respiration. *Glob. Chang. Biol.* **16**, 1366–1379 (2010).

41. Leng, L. *et al.* Biochar stability assessment by incubation and modelling: Methods, drawbacks and recommendations. *Sci. Total Environ.* **664**, 11–23 (2019).

42. Kögel-Knabner, I. *et al.* Biogeochemistry of paddy soils. *Geoderma* **157**, 1–14 (2010).

43. Masiello, C. A. & Druffel, E. R. M. Black Carbon in Deep-Sea Sediments. *Science (80-. ).* **280**, 1911–1913 (1998).

44. Kuzyakov, Y., Bogomolova, I. & Glaser, B. Biochar stability in soil: Decomposition during eight years and transformation as assessed by compound-specific 14C analysis. *Soil Biol. Biochem.* **70**, 229–236 (2014).

45. Dharmakeerthi, R. S., Hanley, K., Whitman, T., Woolf, D. & Lehmann, J. Organic carbon dynamics in soils with pyrogenic organic matter that received plant residue additions over seven years. *Soil Biol. Biochem.* **88**, 268–274 (2015).

46. Fang, Y. *et al.* Interactive carbon priming, microbial response and biochar persistence in a Vertisol with varied inputs of biochar and labile organic matter. *Eur. J. Soil Sci.* **70**, 960–974 (2019).

47. Fang, Y., Singh, B. P. & Singh, B. Temperature sensitivity of biochar and native carbon mineralisation in biochar-amended soils. *Agric. Ecosyst. Environ.* **191**, 158–167 (2014).

48. Kuzyakov, Y., Subbotina, I., Chen, H., Bogomolova, I. & Xu, X. Black carbon decomposition and incorporation into soil microbial biomass estimated by 14 C labeling. *Soil Biol. Biochem.* **41**, 210–219 (2009).
